# Relationship between CMR derived myocardial extracellular volume and myocardial replacement scarring in non-ischemic cardiomyopathy

**DOI:** 10.1186/1532-429X-16-S1-P326

**Published:** 2014-01-16

**Authors:** Mohamad G Ghosn, Kongkiat Chaikriangkrai, Sherif F Nagueh, Dipan J Shah

**Affiliations:** 1 Houston Methodist DeBakey Heart & Vascular Center, Houston Methodist Research Institute, Houston, Texas, USA; 2 Medicine, Houston Methodist Hospital, Houston, Texas, USA; 3 Cardiology, Houston Methodist Research Institute, Houston, Texas, USA

## Background

The measurement of the extent of myocardial replacement fibrosis in non-ischemic cardiomyopathy (NICMP) has been shown to correlate with clinical cardiac outcomes Although delayed enhancement cardiovascular magnetic resonance (DE-CMR) has been established as the reference standard for assessing myocardial replacement fibrosis (RF), this imaging process is unable to identify more subtle diffuse interstitial fibrosis (IF) in the myocardium. Newer T1 mapping after the administration of gadolinium contrast has been shown to adequately assess increases in extracellular volume (ECV) fraction, which occurs in the setting of IF. In this study, we sought to evaluate the relationship between RF and IF in patients with NICMP.

## Methods

Eighty consecutive patients with NICMP underwent DE-CMR and T1 mapping using a Modified Look-Locker Inversion recovery (MOLLI) technique. RF extent was determined by analysis of the DE-CMR images. The extracellular gadolinium contrast agent in the myocardium was compared to the amount of contrast in the blood in the dynamic steady state in order to estimate the ECV fraction. In order to decrease statistical variance, regions of localized RF detected on imaging were avoided when measuring the ECV.

## Results

There was RF evident in 49 patients (61%). Patients with RF had higher ECV compared to those without RF (36.1 ± 10.7 and 31.9 ± 6.7 respectively; p = 0.04). The left ventricular ejection fraction (LVEF) and stroke volume (SV) were significantly lower in patients with ECV > 30% (p < 0.005). The same relation held true for patients with RF (p < 0.005). In the RF group, the mean scar burden was 7.4 ± 9.9% of left ventricle myocardium. In patients without RF, those with ECV > 30 has significantly lower LVEF (p = 0.02). There was a significant increase in ECV for patients with RF of more than 5% of LV myocardium (p < 0.001). In patients without RF, ECV was calculated to be 31.88 ± 6.70% whereas patients with RF between 1-5% and > 5% had ECV of 32.60 ± 6.20 and 44.70 ± 13.91%, respectively. In linear regression analysis, ECV is positively associated with scar burden (Beta weight = 0.527, p < 0.001).

## Conclusions

The results of this study suggest that there is a positive relationship between increased RF and ECV in patients with NICMP. Our data shows that stroke volume and left ventricular ejection fraction are significantly decreased in patients with RF and in those with an increased ECV.

## Funding

None.

